# Thyrotroph Embryonic Factor Regulates Light-Induced Transcription of Repair Genes in Zebrafish Embryonic Cells

**DOI:** 10.1371/journal.pone.0012542

**Published:** 2010-09-07

**Authors:** Daria Gavriouchkina, Sabine Fischer, Tomi Ivacevic, Jens Stolte, Vladimir Benes, Marcus P. S. Dekens

**Affiliations:** Genomics Core Unit, European Molecular Biology Laboratory, Heidelberg, Federal Republic of Germany; Texas A&M University, United States of America

## Abstract

Numerous responses are triggered by light in the cell. How the light signal is detected and transduced into a cellular response is still an enigma. Each zebrafish cell has the capacity to directly detect light, making this organism particularly suitable for the study of light dependent transcription. To gain insight into the light signalling mechanism we identified genes that are activated by light exposure at an early embryonic stage, when specialised light sensing organs have not yet formed. We screened over 14,900 genes using micro-array GeneChips, and identified 19 light-induced genes that function primarily in light signalling, stress response, and DNA repair. Here we reveal that PAR Response Elements are present in all promoters of the light-induced genes, and demonstrate a pivotal role for the PAR bZip transcription factor Thyrotroph embryonic factor (Tef) in regulating the majority of light-induced genes. We show that *tefβ* transcription is directly regulated by light while transcription of *tefα* is under circadian clock control at later stages of development. These data leads us to propose their involvement in light-induced UV tolerance in the zebrafish embryo.

## Introduction

The daily sunlight-darkness cycle is one of the most extreme and repetitive variations in environmental conditions that organisms are exposed to. The necessity to adapt gave rise to light detection mechanisms and a circadian clock, which times a variety of physiological and cellular processes. Several circadian components and DNA damage response proteins are closely related. For example the Cryptochrome (Cry) proteins that transduce the light signal to the circadian clock, either as photoreceptors or as transcriptional repressors, belong to the same family of flavin-containing proteins as the DNA repair enzyme Photolyase (Phr) [1]. Photolyase may have been the first existing light-detecting molecule [2]. Pittendrigh [3], and thereafter Gehring and Rosbash [4], proposed that a circadian oscillator was established and coupled to these blue light photoreceptors to anticipate damage. Interestingly, a link between the clock and nucleotide excision repair was recently reported [5]. Thus light detection and the circadian clock may have originated to avoid DNA lesions [6]. How the light signal is detected in cells, and how seemingly independent processes are integrated with the circadian clock remains unresolved.

In mammals a centralised clock, which resides in the hypothalamic suprachiasmatic nucleus (SCN) and is innervated by the retina, controls temporal adaptation. This master clock relays a signal to the peripheral clocks thereby setting their phase. Zebrafish rely on peripheral circadian clocks that are directly entrained by light, indicating a high degree of cell autonomy [7], [8]. The core clock mechanism consists of a self-sustained transcription-translation auto-regulatory feedback loop [9]. The heterodimer composed of Clock (Clk) and Brain muscle ARNT-like (Bmal) binds to enhancers upstream of the *period* (*per*) and *cryptochrome* (*cry*) genes to initiate their transcription. The repressors Per and Cry interact with the Clk:Bmal heterodimer and thereby down-regulate their own expression.

In zebrafish light-induced activation of the mitogen-activated protein kinase (MAPK) pathway has been shown to regulate *per2*, *cry1a* and *64phr* expression [10], [11]. Thus the same light-signalling pathway controls light dependent UV tolerance and circadian clock entrainment. Also several basic leucine-zipper (bZip) transcription factors play a role in mediating the regulation of light dependent processes. The AP-1 (Activator Protein-1) complex, a heterodimeric protein composed of the bZip transcription factors c-Fos and c-Jun, exhibits light dependent transcription. The transcription factor AP-1 is regulated by the MAPK signal transduction pathway, and is an important component of the mammalian UV response [12]–[14]. Furthermore, TEF (Thyrotroph Embryonic Factor), DBP (D-site Binding Protein) and HLF (Hepatocyte Leukaemia Factor), belonging to the proline- and acidic amino acid-rich (PAR) bZip subfamily, mediate the regulation of metabolic detoxification and are under circadian clock control in mouse [15]. These transcription factors transactivate target genes by binding as homo or heterodimers to the PAR Response Elements (PARRE) in their promoters [16]. However, signal-induced gene expression is not mediated by linear signal transduction pathways targeting a single response element, but involves networks of signalling molecules and transcription factors targeting multiple control elements that cooperate to regulate gene transcription.

This study aims to attain insight into the light signalling mechanism by screening for genes that are light activated and subsequently identifying common regulatory networks driving these genes. The zebrafish is a particularly suitable organism for studying light dependent transcription due to the ability of each cell to directly detect light. We analysed over 14,900 transcripts using the Affymetrix micro-array GeneChip and identified 19 genes that exhibit light-induced transcription. Here we demonstrate by computational promoter analysis in combination with knock down experiments that the PAR bZip transcription factor Tef plays a key role in the regulation of light-induced genes that function primarily in DNA repair and in counteracting the adverse effects of reactive oxygen species.

## Results and Discussion

### A screen for genes that display light dependent transcription

**Figure 1:**
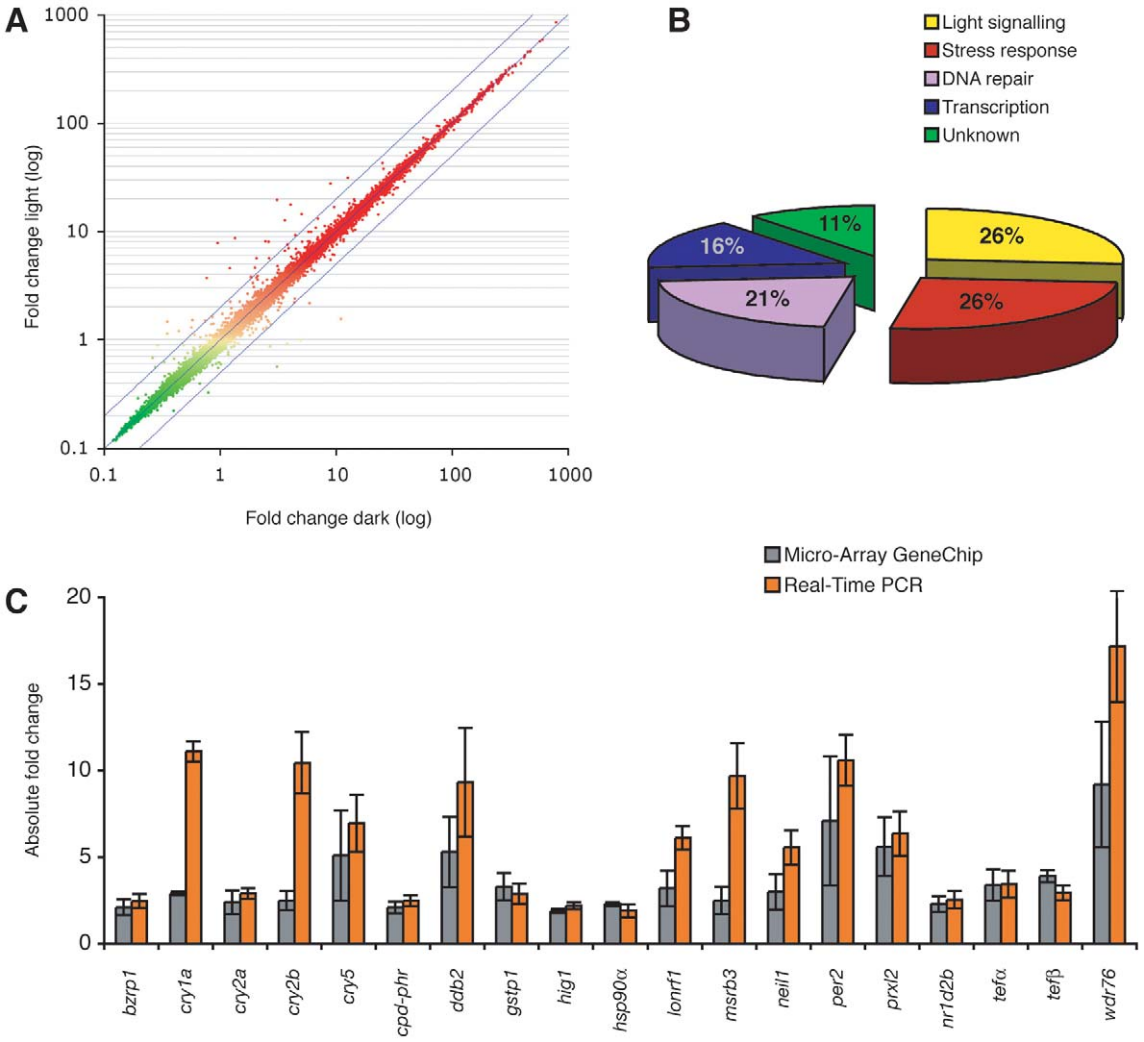
Screen for genes that display light-induced transcription. (A) Scatter plot showing the fold change in transcript level between embryos (9 h.p.f.) that were exposed to light and siblings maintained in darkness. Each dot represents one transcript of 14,900 genes screened. The reliability of the data is indicated by a green to red colour scale, with only the red dots representing transcripts that have a trustworthy differential expression. The outer blue lines demarcate the 2.0 fold boundaries when related to the average (central blue line). (B) Pie chart representing the ratios of the different processes in which light-induced genes function. (C) Validation by qPCR (n = 7) of the 19 light-induced transcripts that were identified by the Affymetrix micro-array GeneChip (n = 3, fold change >2). Grey bars indicate micro-array fold change and orange bars indicate qPCR fold change. Error bars indicate the standard deviation in all experiments. The qPCR fold changes shown were normalised using DD transcript levels, and differences between samples were corrected with β-actin mRNA levels.

Zebrafish embryos become light responsive around 5 hours post fertilisation (h.p.f.), and light sensitivity increases during the following 4 hours [17], [18]. The far from fully differentiated cells at this early stage of development are an appealing model as one can select for genes that display light dependent transcription before specialised light sensing organs have formed. We screened with the Affymetrix micro-array GeneChip for light regulated genes by comparing the differential expression between embryos exposed for the first 9 hours of development to light (LL) and siblings maintained in constant darkness (DD) (Figure 1A, Table 1). All genes that have a differential expression of 2-fold or more on the micro-array chip were validated by quantitative Polymerase Chain Reaction (qPCR) (Table 1, Figure 1C), thereby confirming 19 transcripts to be significantly induced by light in zebrafish early embryonic cells. Interestingly, this light-induced gene set shows no similarity with light-induced transcripts in the mouse SCN [14], the specialized direct light responsive cells in a mammal. Furthermore, the genes that are suppressed by light, apart from the circadian clock regulated gene *egln3* [19], could not be reproduced by qPCR (Table S1). The light-induced genes identified function in DNA repair, stress response, and light signalling (Figure 1B, Table 1).

**Table 1:** Genes that display light-induced transcription.

Gene Symbol	Gene Name	GeneChip Fold Change	Real-Time PCR Fold Change	Process	GenBank No.
*wdr76*	WD40-repeat protein 76	9.2±3.6	17.2±3.2	DNA repair	XM_693494
*cry1a*	cryptochrome 1a*	2.9±0.1	11.1±0.6	Light signalling	NM_131789
*per2*	period 2 [35], [36]	7.1±3.7	10.6±1.5	Light signalling	NM_182857
*cry2b*	cryptochrome 2b	2.5±0.6	10.5±1.8	Light signalling	NM_131792
*msrb3*	methionine sulfoxide reductase B3	2.5±0.8	9.7±1.9	Stress response	NM_001002094
*ddb2*	UV damage DNA binding protein 2	5.3±2.0	9.3±3.1	DNA repair	NM_001083061
*cry5*	cryptochrome 5 [17]	5.1±2.6	7.0±1.6	Light signalling	NM_131788
*prxl2*	peroxiredoxin-like 2	5.6±1.7	6.4±1.3	Unknown	NM_213313
*lonrf1*	LON-protease ring finger 1	3.2±1.0	6.1±0.7	Unknown	XM_684170
*neil1*	nei endonuclease VIII-like 1	3.0±1.0	5.6±1.0	DNA repair	NM_200283
*tefα*	thyrotroph embryonic factor α	3.4±0.9	3.4±0.8	Transcription	NM_131400
*gstp1*	glutathione S-transferase p1	3.3±0.8	2.9±0.6	Stress response	NM_131734
*tefβ*	thyrotroph embryonic factor β	3.9±0.3	2.9±0.4	Transcription	U96848
*cry2a*	cryptochrome 2a	2.4±0.7	2.9±0.3	Light signalling	NM_131791
*bzrp1*	benzodiazepine receptor 1	2.1±0.5	2.5±0.4	Stress response	NM_001006032
*cpd-phr*	cpd-photolyase-like	2.1±0.3	2.5±0.3	DNA repair	NM_201064
*nr1d2b*	nuclear receptor 1D2b	2.3±0.5	2.5±0.5	Transcription	NM_131065
*hig1*	hypoxia induced gene 1	1.9±0.1	2.2±0.2	Stress response	NM_200100
*hsp90α*	heat shock protein 90α	2.3±0.1	1.9±0.4	Stress response	NM_001045073

*Note that the light-induced gene products could function in several different processes, for instance Cry1a also plays a role in stress response [24].

A large proportion of the light-induced genes belong to the family of Cryptochromes. Many responses to light are mediated by CRYs [20], [21], a subset acts as photopigments [10] while others play a role in light signalling or may take part in the core circadian oscillator, as is the case in mammals. Seven *cry* genes have so far been reported in zebrafish [22]. In particular, zebrafish Cry1a has been shown to reset the clock [23], and also has been reported to play a role in oxidative stress response [24].

Several genes with a function in DNA repair are expressed at high levels in light exposed embryos, including the nucleotide excision repair gene *ddb2* and its homologue *wdr76*. In humans the WDR76 protein is associated with the CUL4-DDB1 ubiquitin ligase complex [25]. Interestingly, an increase in *WDR76* expression is observed during DNA replication [26], which is under circadian clock control in zebrafish [27], [12]. We also observed an increase in transcript level of the DNA glycosylase Neil1, which initiates the first step in base excision repair by cleaving bases damaged by oxygen radicals [28]. Furthermore we demonstrate light-induction of a photolyase-like gene, *cpd-phr*, that removes cyclobutane pyrimidine dimers [29]. *ap-1* is light induced in adult zebrafish [12], however it was not detected by the micro-array chip at an early stage of development.

Reactive oxygen species have been reported to induce the transcription of direct light responsive genes in zebrafish [24]. We show here that the expression levels of many stress response genes are elevated during light exposure, such as *msrb3*, which has a function in the repair of oxidized proteins [30], and *gstp1*, which has a catalytic function in the detoxification of electrophiles thereby neutralizing products of reactive oxygen species [31]. The light-induced peripheral *bzrp1* gene opposes apoptosis during oxidative stress by controlling mitochondrial membrane permeability [32]. Also the stress response gene *hig1* has an anti-apoptotic function [33]. Furthermore we observe higher transcript levels of *hsp90α*, its gene product being essential for refolding of denatured proteins [34].

**Figure 2:**
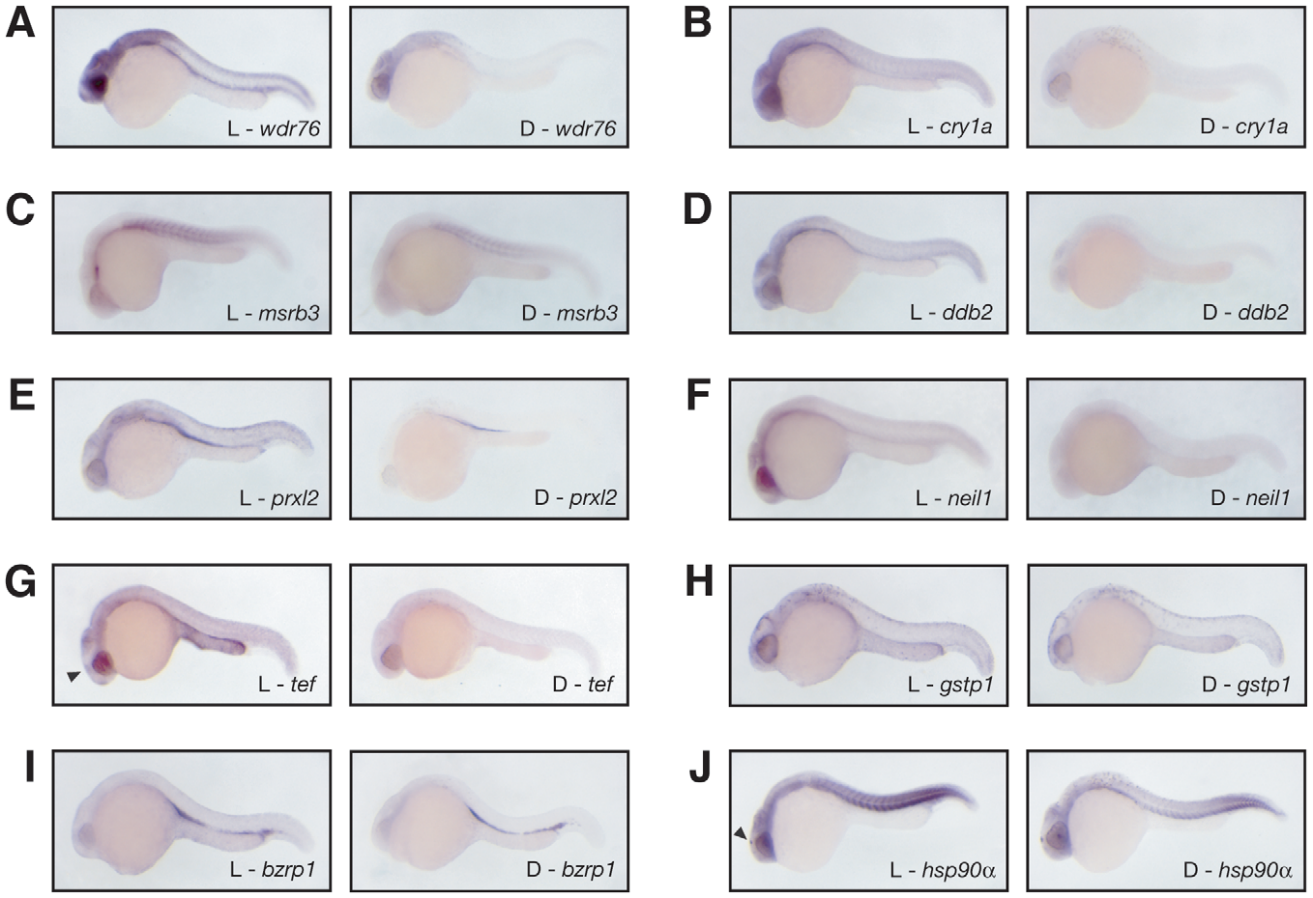
Light-induced transcripts are expressed ubiquitously. (A) In situ hybridizations of embryos (at 25 h.p.f.) that were exposed to light (left) or maintained in darkness (right) for the probe against wdr76, (B) cry1a, (C) msrb3, (D) ddb2, (E) prxl2, (F) neil1, (G) tef, (H) gstp1, (I) bzrp1, and (J) hsp90α. All light-induced transcripts are expressed ubiquitously at the early stages of zebrafish development. For most transcripts a gradient is observed with the highest level of expression at the anterior. The wdr76, cry1a, msrb3, tef, and neil1 transcripts are present at substantially higher levels in the retina. The hsp90α and tef transcripts show a distinctive presence in the pineal (indicated by arrowhead).

We applied *in situ* hybridization to several of the light-induced genes during retinal development to determine if any of the genes display enriched expression in specialized light sensing tissues (Figure 2A–J). Published *in situ* patterns of light-induced transcripts were not examined [17], [35], [36]. All transcripts are ubiquitously expressed, and the transcripts of *wdr76*, *cry1a*, *msrb3*, *neil1*, and *tef* show higher levels in the retina, and the *hsp90α* and *tef* transcripts are present at a higher level in the pineal (Figure 2A, 2B, 2C, 2F, 2G, 2J).

**Figure 3:**
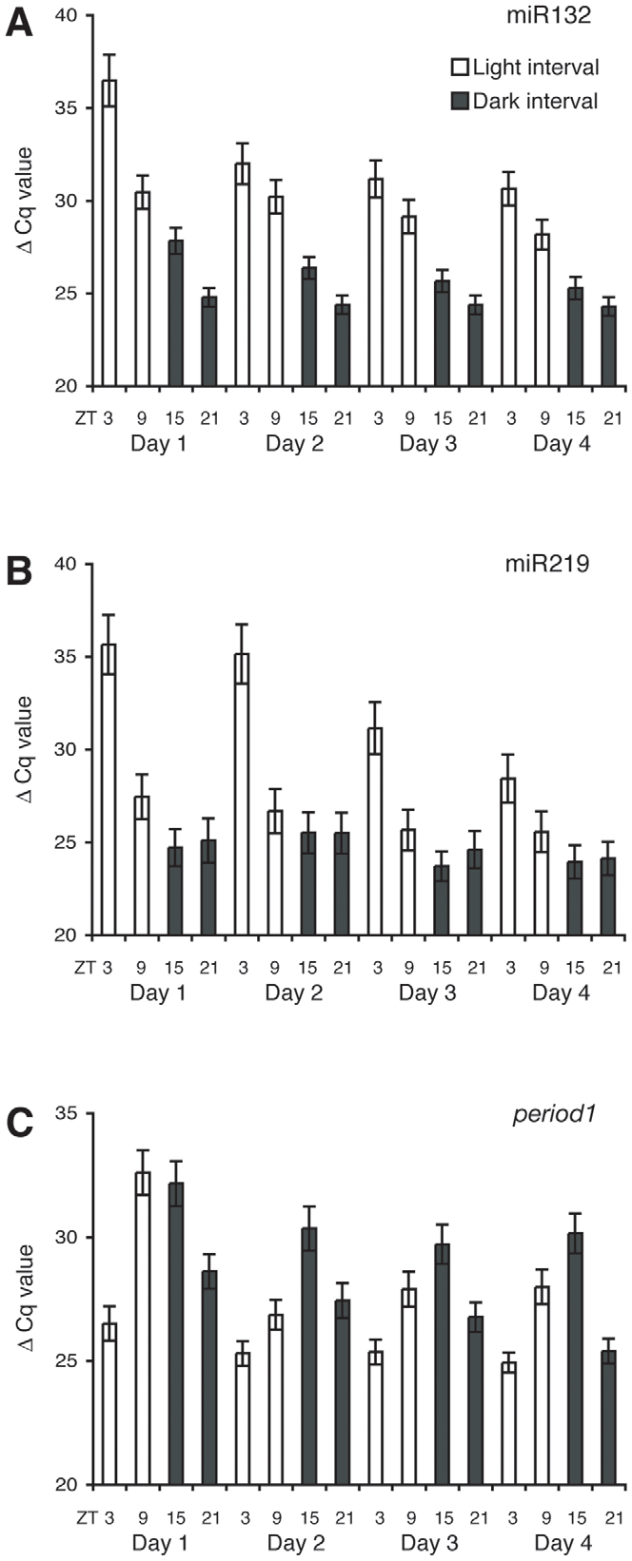
miR219 and miR132 temporal expression pattern. (A) qPCR analysis showing the temporal oscillation of miR132 and (B) miR219 transcription during the first four days of development in embryos raised under a 12∶12 LD cycle. (C) Expression of *period1* under the same conditions. White bars indicate the light and black bars the dark intervals. Note that low cycle quantification (Cq) values indicate high transcript levels and vice versa.

The micro RNAs (miRNAs), a class of small non-coding transcripts, play a key role in post-transcriptional gene regulation. Since the zebrafish genome array GeneChip did not include the detection of miRNAs, we extended the screen by testing reported oscillating mouse and *Drosophila* miRNAs for rhythmic expression in zebrafish. Animals were entrained on light-dark (LD) cycles and samples were taken during the first four days post fertilisation. We show by qPCR that miR132 and miR219 are rhythmically expressed during zebrafish development (p<0.05; Figure 3A and 3B). Interestingly, the miRNAs show peak and trough transcript levels at opposite zeitgeber times (zt) as reported for mouse [37].

### Tef mediates the regulation of light-induced transcription

**Figure 4:**
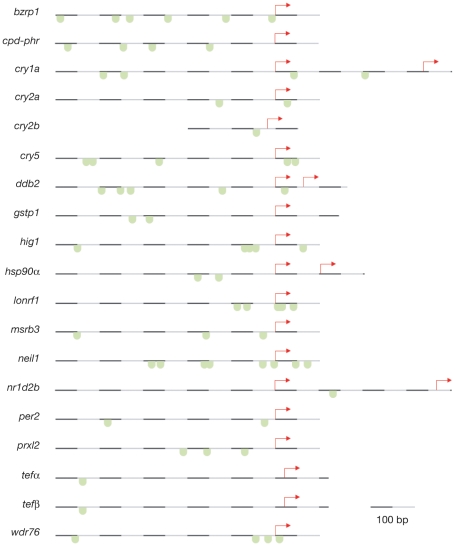
All promoters of the light-induced genes contain PAR elements. Genomatix software was used to predict PAR response elements in a 500 bp region upstream of the start codon in the promoters of all light-induced genes. Since the untranslated region of *cry2b* is not present in the Genomatix database this gene was analysed separately. Start codons are indicated with red arrows, and the locations of predicted PAR elements are marked with green boxes.

Given that Tef and Nr1D2b are the only transcription factors identified by the screen, we hypothesized whether they could play a central role in the activation of light-induced transcription. The *tef* gene encodes a PAR bZip transcription factor that binds to PARREs (PAR Response Elements) in the promoters of target genes, and is under circadian clock control in mouse [38]. The *nr1d2b* (*rev-erbβ*) gene encodes an orphan nuclear receptor that binds to ROR (RAR-related Orphan Receptor) elements in the promoters of target genes, and is a paralog of a regulatory component of the circadian clock (*nr1d1* or *rev-erbα*) [39]. To further investigate their role, we used phylogenetic promoter analysis software (GenomatixSuite) to screen for regulatory sequences in the light-induced gene set. This predicted the presence of PAR response elements (puTTApyGTAApy) in the promoters of all light-induced genes (Figure 4), however only a few promoters include E-box and ROR elements. Since all promoters contain PARREs, this points to Tef being an evident candidate for the regulation of light-induced transcription.

**Figure 5:**
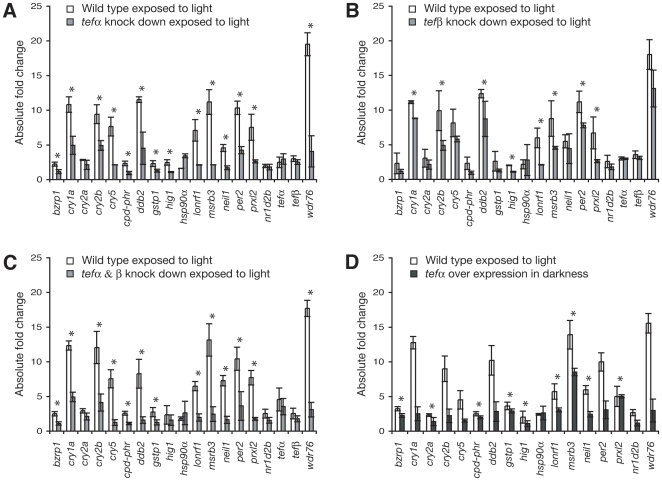
Tef regulates light-induced transcription. (A) Transcript level analysis by qPCR after 9 hours of light exposure in morpholino microinjected *tef*α knock down embryos (grey bars) compared to untreated embryos (white bars). Asterisks indicate significant difference in expression level. (B) Experiment as in A for morpholino-mediated knock down of *tefβ* in light exposed embryos. Demonstrating the reduced effect of *tefβ* knock down on the levels of most light-induced transcripts when compared with *tef*α knock down. (C) Morpholino-mediated double knock down of *tef*α and *tefβ* in light exposed embryos matches the *tef*α knock down result. (D) Embryos microinjected with *tef*α mRNA and directly transferred to DD. *tef* over expression results in elevated transcript levels when compared to untreated embryos maintained in DD. Fold changes were normalized with DD transcript levels, thus the knock down, over expression, and wild type light exposed transcript levels are compared to the wild type expression level in DD, which is set at zero on the Y-axis within each bar. Differences between samples were corrected with *β-actin* mRNA levels. These data clearly demonstrate that Tef mediates the regulation of light-induced transcription.

To verify the computational data, we next tested whether knock down of *tef*, by injecting morpholino-modified anti-sense oligonucleotides in light exposed embryos, reduces the transcript levels of light-induced genes. Two zebrafish isoforms have been reported, *tefα* and *tefβ*, which are transcribed from separate promoters [40]. Knock down of *tefα* results in strongly reduced transcript levels of: *bzrp1*, *cpd-phr*, *cry1a*, *cry2b*, *cry5*, *ddb2*, *gstp1*, *lonrf1*, *msrb3*, *neil1*, *per2*, *prxl2*, and *wdr76* (p<0.05; Figure 5A). Interestingly, a zebrafish *per2* promoter study showed this gene to be regulated by Tef [41], and thus supports the data presented here. Furthermore, a reduced *gstp1* transcript level has been reported in *Hlf*/*Dbp*/*Tef* triple knock out mice [15]. *tefα* knock down does not significantly affect miR132 and miR219 expression levels, consistent with the absence of PARREs in their promoters (data not shown). Knock down of *tefβ* mildly reduced expression of: *cry1a*, *ddb2*, *hig1*, *per2*, and strongly reduced expression of: *cry2b*, *lonrf1*, *msrb3*, and *prxl2* (p<0.05; Figure 5B). Double knock down of *tefα* and *tefβ* produces the same effect as single *tefα* knock down, but results in even lower levels of *cry2b* and *ddb2* (p<0.05; Figure 5C). The reduced transcript level in *tef* knock down embryos is consistent with direct regulation by transcriptional activation, as suggested by the PARREs present in the promoters of the light-induced genes. However, it cannot be ruled out that Tef indirectly regulates these genes. Since the transcript levels of light regulated genes in *tef* knock down LL embryos are rarely reduced to their DD levels, other factors must also play a role in controlling light dependent processes. Importantly, several other members of the bZip family have the capacity to bind the PARRE. In all knock down experiments, *tef* mRNA levels are not affected, implying that *tef* does not regulate itself although PARREs are present in the *tef* promoters. To confirm the knock down data we over expressed *tefα* by microinjecting mRNA in DD embryos, this results in a significant increase in the levels of: *bzrp1*, *cpd-phr*, *cry2a*, *gstp1*, *hig1*, *lonrf1*, *msrb3*, *neil1*, and *prxl2* (p<0.05; Figure 5D). Thus we demonstrate *tefα* to play a crucial role in the regulation of many light-induced genes, while *tefβ* has a less prominent function.

### Tefα is under circadian clock control

**Figure 6:**
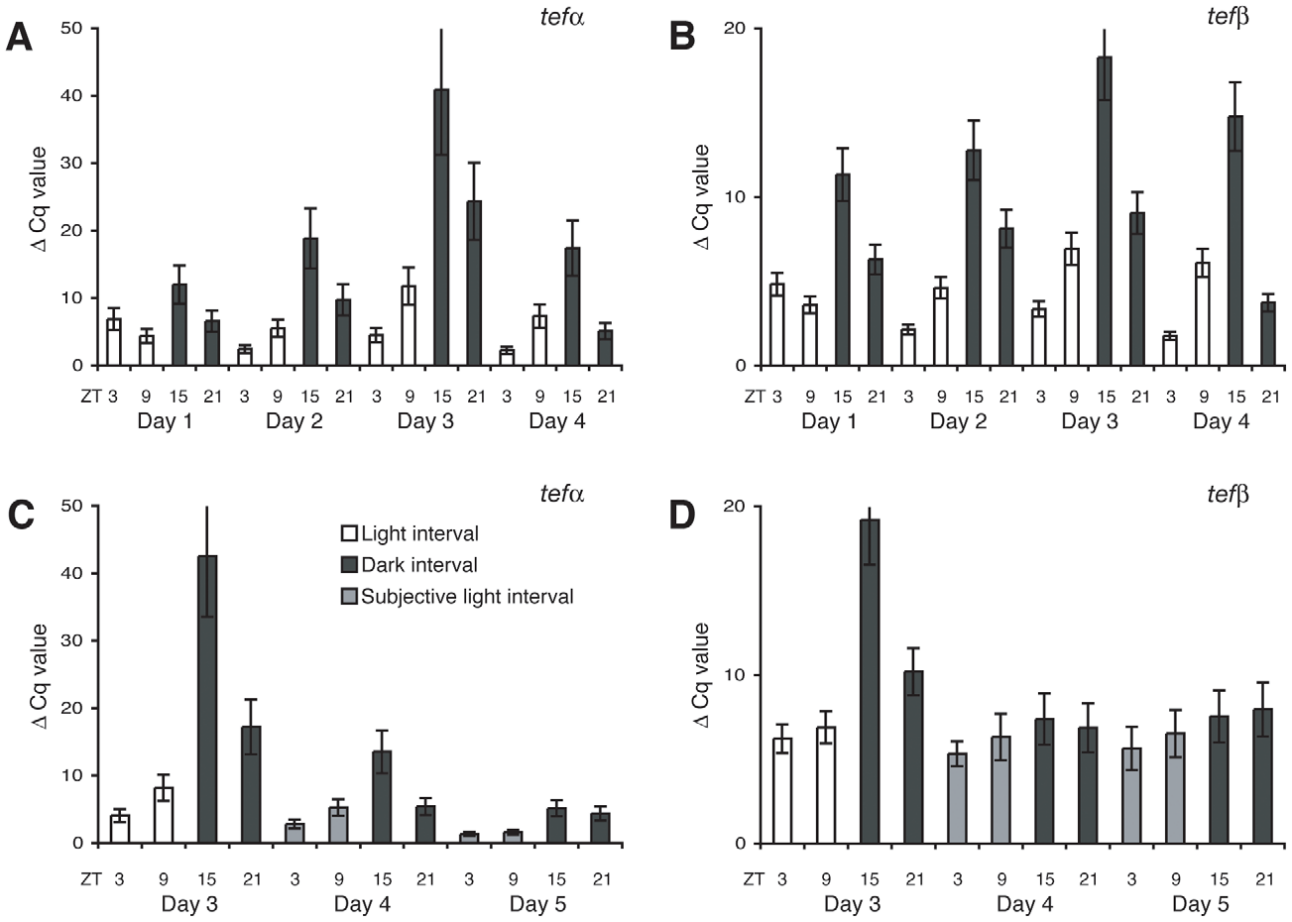
Transcription of *tef*α is under circadian clock control. (A) qPCR analysis showing the temporal transcript levels of *tef*α and (B) *tefβ* during the first four days of development in embryos raised under a 12∶12 LD cycle. White bars indicate the light and black bars the dark intervals. (C) *tef*α mRNA levels on days 3, 4 and 5 in embryos entrained to LD cycles for the first 3 days followed by DD. Grey bars indicate the subjective light interval. The continuation of rhythmic expression in DD demonstrates that *tef*α transcription is regulated through an oscillator. (D) *tefβ* mRNA levels under the same experimental conditions as C. In all experiments the differences between samples were corrected with *β-actin* mRNA levels. Note that low Cq values indicate high transcript levels and vice versa.

Based on the regulation of *tef* in mouse, we hypothesised whether the two isoforms of Tef are under circadian clock control. We entrained zebrafish larvae to LD cycles for the first four days of development and analysed *tef* mRNA levels. This revealed that *tefα* and *tefβ* transcription oscillate during development, showing peak and trough transcript levels at opposite zt's as reported for mouse [42]. The *tef* transcript level reaches its peak during the light period, as expected from its role in mediating the regulation of light-induced transcription (Figure 6A and 6B, note that low cycle quantification values indicate high transcript levels and vice versa). To investigate if *tef* is under circadian clock control, larvae were subjected to LD cycles during the first 3 days of development followed by DD over the consecutive days. A rhythm of *tefα* transcription is observed on the days following the LD cycles (Figure 6C). The observed light entrainment reflects *tefα* regulation through an oscillator. As it is currently technically not feasible to determine how *tefα* is regulated at the earliest stages of development, the possibility exists that *tefα* is initially directly induced by light. The difference in *tefβ* transcript levels is not significant on the first and second day in DD (p>0.05; Figure 6D), thus suggesting a direct light driven mechanism during the first days of development, however this gene may later on become under circadian clock control. The role for Tefα in the regulation of stress response and DNA repair genes may imply the circadian clock in their regulation. Gachon and colleagues [15] demonstrated a role for the circadian transcription factors TEF, DBP, and HLF in the regulation of various processes in mouse, including metabolic detoxification. TEF, DBP, and HLF are expected to regulate different target genes as they have different target promoter preferences [38], [42]. Triple knock out of all PAR bZip family members in mouse results in epilepsy and accelerated ageing, but does not lead to developmental defects [43]. In the mouse embryo *Tef* expression is only present in the anterior pituitary [44], while in the zebrafish embryo it is ubiquitously transcribed. This may imply centralized regulation during development for the mouse *Tef* gene in contrast to the zebrafish embryo.

### Light-induced UV tolerance

Zebrafish spawn at light onset in shallow rivers, thus the embryos are already exposed to sunlight at the earliest stage of development. Here we demonstrate that visual light induces genes that function in light signalling, stress response or DNA repair in zebrafish embryonic cells. We used a light source that only emits low radiation in the UV-A and no radiation in the harmful UV-B and UV-C range of the spectrum (Figure S1), and the embryos were separated from the source by a 4 mm Perspex plate and at least 5 cm water. Therefore it is not possible that the identified DNA repair genes were induced by UV damage. Interestingly, embryos maintained in constant darkness and subsequently UV-irradiated show a lower survival rate when compared to UV-treated embryos that were previously exposed to light [17]. The superior survival in the latter case can be explained by the increase in transcription of stress response and DNA repair genes. Several mechanisms that relay the light signal directly or via the circadian clock can be envisaged. Light-induced activation of the MAPK signalling pathway plays an important role in 64Phr expression [11], which has the capacity to repair DNA in zebrafish. Cry1a can convey the light signal to the circadian clock as it binds directly to the core clock components Clk and Bmal [23], [45]. We demonstrate *tefβ* expression to be directly driven by light in the zebrafish embryo, and *tefα* transcription to be under circadian clock control at later stages of development. Although the *tef* genes seem to be differently regulated, it is most likely that both *tef* genes are initially directly regulated by light and later on become under the control of the circadian clock. In addition, it is plausible that *tef* is regulated through the circadian clock as well as a direct light pathway. Since more DNA lesions are induced when cells are exposed to sunlight than during the night, the number of mutations could most likely be reduced if DNA damage were anticipated. Indeed DNA excision repair was demonstrated to be under circadian clock control in mammals [5]. We show here that the Tef transcription factors play a pivotal role in regulating DNA damage and stress response processes in the zebrafish embryo, and we suggest their involvement in light-induced UV tolerance. At later stages Tef could play a key role in coupling the circadian clock to repair processes, as several of the light-induced repair genes are rhythmically transcribed during development. Considering its crucial function in regulating various processes, ranging from metabolic detoxification to DNA repair, this transcription factor will be of high interest for future research.

## Materials and Methods

### Experimental setup

Zebrafish were raised following standard protocols [46]. Embryos were transferred to tissue culture flasks and submerged in thermostatically controlled water baths to maintain a constant temperature of 28°C. The setup is positioned within a light-sealed and air conditioned box. Embryos were illuminated with a compact fluorescent lamp (140 µW/cm^2^, Figure S1) connected to a timer. The spectrum of the light source was determined using a fiber optic spectrometer (USB2000, OceanOptics Inc).

### Micro-array hybridization assay

Differential gene expression was determined by comparing two conditions: one group of sibling embryos was exposed to light, while the other group of siblings was transferred to constant darkness within 30 min after being laid. Embryos were harvested at 9 h.p.f. (ZT/CT9), and total RNA was extracted from 50 embryos per group using TRIzol Reagent (Gibco BRL) according to the manufacturer's instructions. From each condition 3 µg total RNA was used to synthesize biotinylated cRNA according to the one-cycle protocol, followed by hybridization to the Affymetrix GeneChip Zebrafish Genome Array. All procedures were conducted using Affymetrix equipment, protocols, and GeneChip Operating Software. GeneSpring GX7.3 software (Agilent Technologies) was used for CEL file data analysis. The MAS5 algorithm was applied for condensation, and median normalization was used. The micro-array result presented is the average of three independent experiments. All data is MIAME compliant and deposited in the MIAME database. Transcripts are considered differentially expressed when the average change was 2 fold or more. GenomatixSuite (Genomatix) software was used for subsequent phylogenetic promoter analysis. The light-induced and light-suppressed gene sets were separately analysed. The analysis was performed with selected promoter elements and limited to the first 500 bps upstream of the start codon.

### Quantitative PCR analysis

cDNA was obtained by transcribing 1 µg of total RNA using QuantiTect reverse transcription components (Qiagen). Absolute levels of transcript were determined with fluorescence based Real-Time PCR using Sybr Green PCR Master Mix and thermocyclers from Applied Biosystems (AB). Primers were designed to generate amplicons that cross exon junctions to eliminate contamination through genomic DNA amplification (Universal Probe Library software from Roche; http://www.roche-applied-science.com/sis/rtpcr/upl/ezhome.html). A list of primer sequences for all transcripts is given in Table S2. qPCR was performed using the following thermal cycling parameters: 95°C for 10 min, followed by 40 two-step cycles of 95°C for 15 sec and 60°C for 1 min. All qPCR reactions were carried out in duplicate so that average cycle quantification values could be obtained. The absolute fold change was determined by normalising the level of transcription with the corresponding level in DD, and both levels were corrected for random errors with the *β-actin* level. The abundance of miRNA was also determined by qPCR. miRNAs were transcribed from 10 ng of total RNA using the TaqMan MicroRNA Reverse Transcript Kit (AB) and the human primers and probes for miR-132 [5′-UAACAGUCUACAGCCAUGGUCG-3′] and miR-219 [5′-UGAUUGUCCAAACGCAAUUCU-3′] (AB), followed by Real-Time PCR using the TaqMan Universal PCR Master Mix (AB) as indicated by the manufacturer. The absolute fold change was calculated using the comparative Δ(ΔCq) method (Relative Expression Software Tool) [47], and for all other experiments ΔCq was applied.The significance of the difference observed between two treatments within one experiment was determined with the Bayesian t-test.

### *In situ* hybridization

*In situ* hybridisations were performed with anti-sense RNA fragments according to standard protocols. Probe synthesis was conducted with the components of the DIG RNA Labelling Kit (Roche). Embryos were fixed and subjected to methanol (Merck) and proteinase K (Roche) steps to enhance probe absorption. Embryos were hybridised with probe at 67°C overnight, followed by washing and labelling with sheep α-DIG AP-coupled Fab fragments (Roche) in 2% blocking reagent (Roche) and 10% goat serum (Sigma). The substrate NBT/BCIP (Roche) in 1 M Tris was used for detection.

### Transient knock down and over expression

Transient knock down of *tefα* or/and *tefβ* was performed by microinjecting zygotes with 0.3 mM morpholino-modified anti-sense oligonucleotide (Gene Tools) [48], designed to match the *tefα* [*tefα*_(AUG)_MO: 5′-CGTGATGGAAATAGGCTTCATGTCC-3′] or *tefβ* [*tefβ*_(AUG)_MO: 5′-CTGAAGACATCTCAGAACGGTTTCA-3′] initiation of translation regions. In the case of double knock down a final concentration of 0.5 mM was used. No significant difference in mRNA level was observed between mock injected and untreated embryos. As a control the ATG region of *tef* was cloned in frame of *egfp* lacking its endogenous start codon. The chimeric *tef-egfp* mRNA was co-injected with the corresponding morpholino. We observed suppression of EGFP expression for both morpholinos (Figure S2). For transient over expression *tefα* was cloned into pCS2+ and synthesis of capped mRNA was performed with the SP6 mMessage mMachine components (Ambion). The transcript was column purified (Qiagen) and 100 pg *tefα* mRNA was microinjected into each zygote.

## Supporting Information

Table S1 Genes that display light suppressed transcription.
(0.03 MB DOC)

Table S2 Exon junction crossing Real-Time PCR primers.
(0.05 MB DOC)

Figure S1 Spectrum of compact fluorescent lamp. (A) Spectrum of the light source that was used for all experiments, showing no emission in the hazardous UV-C (below 280nm), and B (320nm-280nm) class, and minimal emission of least harmful UV-A light (range 400nm-320nm). (B) Experimental setup.
(1.07 MB TIF)

Figure S2 *tef* knock down control experiment. To assess the capability of the morpholino to knock down its target, the ATG region of *tef* was cloned in front of *gfp* lacking its endogenous start codon, and the *tef-gfp* mRNA was co-injected with the morpholino. (A) *tefα*_ATG_-*gfp* expression. (B) *tefα*_ATG_-*gfp* co-injected with corresponding morpholino. (C) *tefβ*_ATG_ *-gfp* expression. (D) *tefβ*_ATG_ *-gfp* co-injected with corresponding morpholino.
(1.74 MB TIF)
