# Supplementary material for: Thyrotroph Embryonic Factor Regulates Light-Induced Transcription of Repair Genes in Zebrafish Embryonic Cells
Source: PLoS One. 2010 Sep 7;5(9):e12542. doi: 10.1371/journal.pone.0012542 (PMC2935359; doi:10.1371/journal.pone.0012542)
Supplement: Table S1 — Genes that display light suppressed transcription. (0.03 MB DOC) [file pone.0012542.s001.doc]

###### Table S1: Genes that display light suppressed transcription.

| **Gene**  **Symbol** | Gene Name^*^ | **GeneChip**  **Fold Change** | **Real-Time PCR**  **Fold Change** | **Process^*^** | **GenBank No.^*^** |
| --- | --- | --- | --- | --- | --- |
| *egln3* | egl nine homolog 3 | 14.4 ± 8.0 | 2.9 ± 0.4 | Apoptosis | NM_213310 |
| *p4ha1* | procollagen-proline 2-oxoglutarate 4-dioxygenase alpha polypeptide I | 11.3 ± 7.0**^*^** | 0 | Metabolism**^*^** | NM_214691**^*^** |
| *pfkfb3*^*^ | 6-phosphofructo-2-kinase/fructose-2,6-biphosphatase 3 | 6.2 ± 4.0**^*^** | 0 | Metabolism**^*^** | NM_213397**^*^** |
| *igfbp1*^*^ | insulin-like growth factor binding protein 1a | 3.7 ± 0.5**^*^** | 0 | Proliferation**^*^** | NM_173283**^*^** |
| *plod1*^*^ | procollagen-lysine 1, 2-oxoglutarate 5-dioxygenase 1a | 3.9 ± 1.5**^*^** | 0 | Metabolism**^*^** | NM_001077742**^*^** |
| *aldoc* | aldolase c fructose-bisphosphate | 3.4 ± 0.5 | 0 | Metabolism | NM_194384 |
| *pgam1* | phosphoglycerate mutase 1a | 2.5 ± 0.5 | 0 | Metabolism | NM_198804 |
| *gadd34* | growth arrest and DNA damage-inducible protein | 2.1 ± 0.3**^*^** | 0 | Apoptosis**^*^** | NM_001082921**^*^** |
| *sumf1* | sulfatase modifying factor 1 | 1.9 ± 0.3 | 0 | Metabolism | NM_001040340 |
| *insig1* | insulin induced gene 1 | 2.2 ± 0.5 | 0 | Metabolism | NM_199869 |
| *gpia* | glucose phosphate isomerase a | 2.0 ± 0.4 | 0 | Metabolism | NM_144763 |
| *wsb1*^*^ | WD repeat and SOCS box-containing 1 | 1.9 ± 0.2**^*^** | 0 | Signalling**^*^** | NM_199633**^*^** |
