# Supplementary material for: Thyrotroph Embryonic Factor Regulates Light-Induced Transcription of Repair Genes in Zebrafish Embryonic Cells
Source: PLoS One. 2010 Sep 7;5(9):e12542. doi: 10.1371/journal.pone.0012542 (PMC2935359; doi:10.1371/journal.pone.0012542)
Supplement: Table S2 — Exon junction crossing Real-Time PCR primers. (0.05 MB DOC) [file pone.0012542.s002.doc]

###### Table S2: Exon junction crossing Real-Time PCR primers.

| **Gene**  **Symbol** | Forward Primer^**^ | **Tm (°C) ^*^** | **Position^*^** | Reversed Primer^**^ | **Tm (°C) ^*^** | **Position^*^** | **Size (nt) ^*^** | **GenBank No.^*^** |
| --- | --- | --- | --- | --- | --- | --- | --- | --- |
| *β-actin* | tcactccccttgttcacaataa | 59 | 34-55 | ggcagcgatttcctcatc | 59 | 93-76 | 60 | NM_131031 |
| *bzrp1* | cggtgcacacaaaattcaac | 60 | 401 - 420 | gggataccaggacaccattg | 60 | 466 - 485 | 85 | NM_001006032 |
| *cpd-phr* | ctcacccatattgcgcttct | 60 | 827 - 846 | tccacctcgagtgatgacag | 59 | 880 - 899 | 73 | NM_201064 |
| *cry1a* | agaggcacctggagaggaag | 60 | 665 - 684 | gagtcgacaggagaggcaac | 59 | 773 - 792 | 128 | NM_131789 |
| *cry2a* | ccccaaacccatggtaaac | 59 | 1398 - 1416 | gtacggaggccagaagtcc | 59 | 1492 - 1510 | 113 | NM_131791 |
| *cry2b* | caaacatcgacaccgacact | 59 | 1647 - 1666 | tggtccgtcaggtcactct | 59 | 1700 - 1718 | 72 | NM_131792 |
| *cry5* | tcctgacccgaggtgactt | 59 | 1088 - 1106 | agcagcagctcctcgaac | 59 | 1137 - 1154 | 67 | NM_131788 |
| *ddb2* | tgaaaaaccagtcaatgctga | 59 | 1051 - 1071 | ccggtttagaccagtcgtatg | 59 | 1125 - 1145 | 95 | NM_001083061 |
| *egln3* | aaaaactggaatgccaagga | 59 | 543 - 562 | cgtaaggtttcccttcagga | 59 | 587 - 606 | 64 | NM_213310 |
| *gstp1* | gattcctggttggtgatcaga | 59 | 494 - 514 | caggagggagaaagcacctt | 60 | 559 - 578 | 85 | NM_131734 |
| *hig1* | gatgagaaaaatcaagcagaatcc | 60 | 97 - 120 | ttgaacagtctgtacgccaca | 60 | 157 - 177 | 81 | NM_200100 |
| *hsp90α* | tacgagcagttctccaaaaaca | 59 | 1501 - 1522 | ctttctgttctgcgagtcttca | 59 | 1539 - 1560 | 60 | NM_001045073 |
| *lonrf1* | aaagtgattccgggcaga | 59 | 788 - 805 | tgacacaggcttagacagcac | 59 | 840 - 860 | 73 | XM_684170 |
| *msrb3* | tccagaatatttgcgtacaaagaa | 60 | 1108 - 1131 | atcccagcaggcacacata | 60 | 1218 - 1236 | 129 | NM_001002094 |
| *neil1* | ctgcaggaaaagcaaaacaa | 59 | 1304 - 1323 | tgaaaagtattctgattgttgagtcac | 59 | 1372 - 1398 | 95 | NM_200283 |
| *nr1d2b* | catgcctgtgagggatgtaa | 59 | 711 - 730 | cagctttcgttcttaaggcact | 60 | 775 - 796 | 86 | NM_131065 |
| *per1* | gtcggatgatgacaaacagc | 59 | 2683 - 2702 | ccagagacacagacggacct | 60 | 2725 - 2744 | 62 | NM_212439 |
| *per2* | ccaacgtggacgaagatgta | 59 | 3851 - 3870 | gcagcaccttctggatgtct | 60 | 3897 - 3916 | 66 | NM_182857 |
| *prxl2* | ggaggcatgctcattgttg | 60 | 494 - 512 | attgtctgcaggcgatttct | 59 | 549 - 568 | 75 | NM_213313 |
| *tefα* | aaggcaataaatgaataatagtttgga | 59 | 1245 - 1271 | tcacctgcttctatcttgtctcc | 59 | 1295 - 1317 | 73 | NM_131400 |
| *tefβ* | gtgcctgaggatcagaagga | 60 | 702 - 721 | gatcgtttggctgcaacat | 59 | 754 - 772 | 71 | U96848 |
| *wdr76* | tgccattgttgaccgtagaa | 60 | 1149 - 1168 | gcacagggtggacatgaac | 59 | 1222 - 1240 | 92 | XM_693494 |
