# Supplementary figures and images for: Thyrotroph Embryonic Factor Regulates Light-Induced Transcription of Repair Genes in Zebrafish Embryonic Cells
Source: PLoS One. 2010 Sep 7;5(9):e12542. doi: 10.1371/journal.pone.0012542 (PMC2935359; doi:10.1371/journal.pone.0012542)

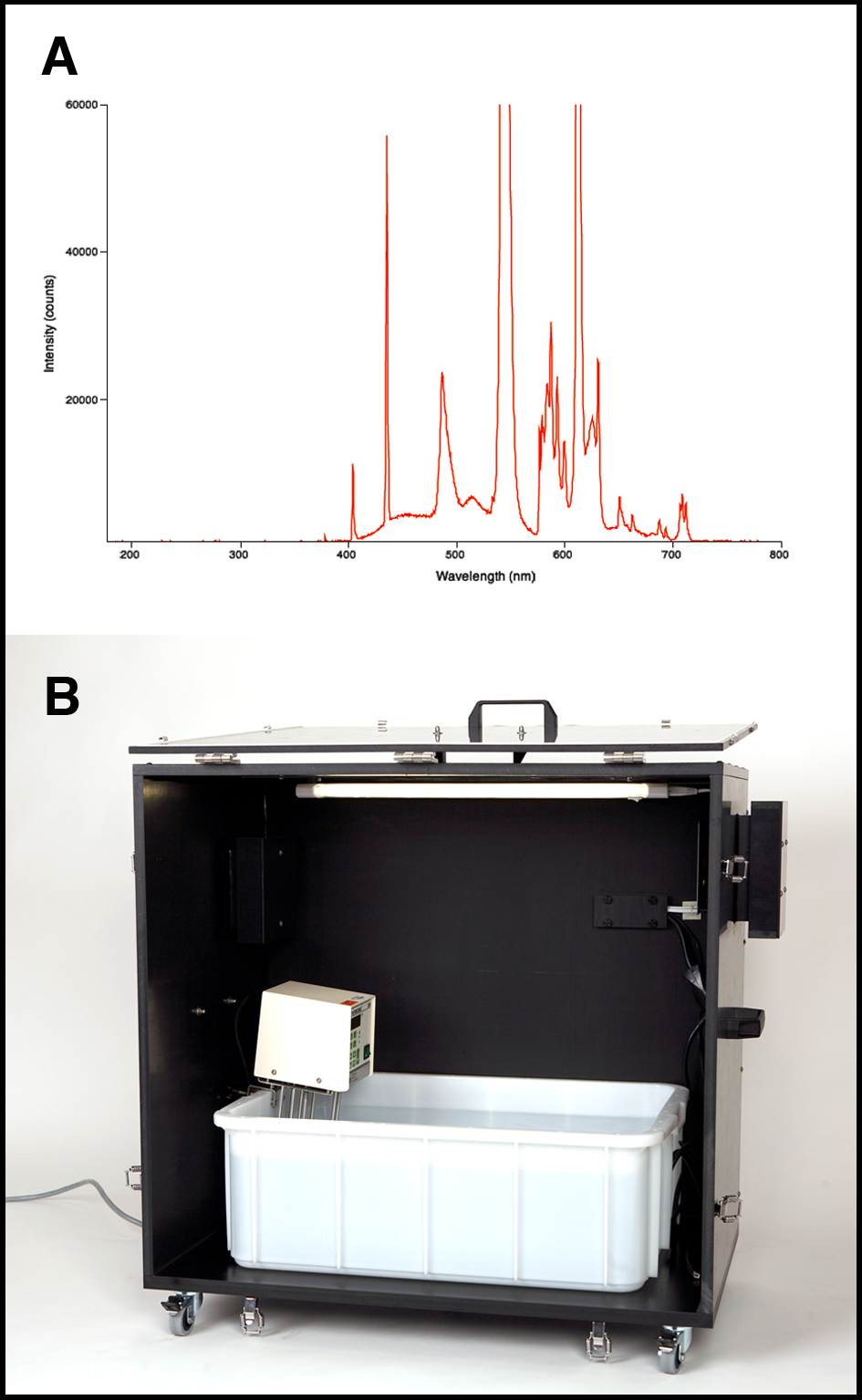

Supplement: Figure S1 — Spectrum of compact fluorescent lamp. (A) Spectrum of the light source that was used for all experiments, showing no emission in the hazardous UV-C (below 280nm), and B (320nm-280nm) class, and minimal emission of least harmful UV-A light (range 400nm-320nm). (B) Experimental setup. (1.07 MB TIF) [file pone.0012542.s003.tif]

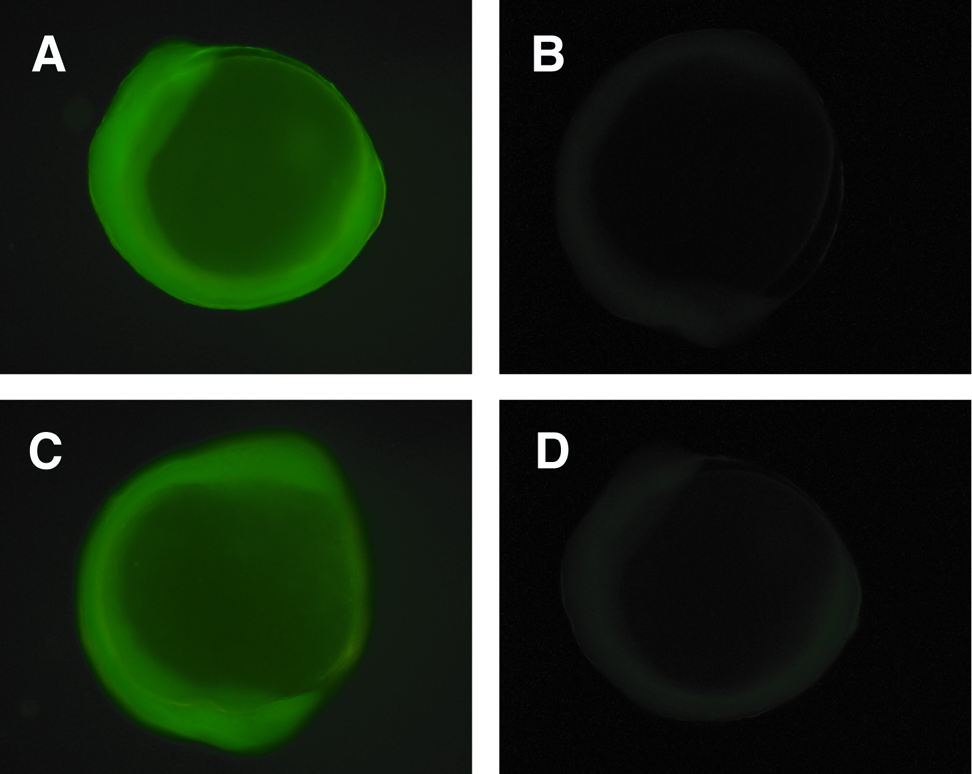

Supplement: Figure S2 — tef knock down control experiment. To assess the capability of the morpholino to knock down its target, the ATG region of tef was cloned in front of gfp lacking its endogenous start codon, and the tef-gfp mRNA was co-injected with the morpholino. (A) tefα ATG-gfp expression. (B) tefα ATG-gfp co-injected with corresponding morpholino. (C) tefβ ATG -gfp expression. (D) tefβ ATG -gfp co-injected with corresponding morpholino. (1.74 MB TIF) [file pone.0012542.s004.tif]
